# Measuring agreement between healthcare survey instruments using mutual information

**DOI:** 10.1186/s12911-016-0335-y

**Published:** 2016-07-26

**Authors:** Yuncheol Kang, Melinda R. Steis, Ann M. Kolanowski, Donna Fick, Vittaldas V. Prabhu

**Affiliations:** 1 Department of Industrial Engineering, Hongik University, Seoul, 04066 Republic of Korea; 2 Orlando Veterans’ Administration Medical Center, Viera, Florida USA; 3 College of Nursing, Pennsylvania State University, University Park, Pennsylvania, USA; 4 Department of Industrial and Manufacturing Engineering, Pennsylvania State University, University Park, Pennsylvania, USA

**Keywords:** Healthcare survey instrument, Agreement, Mutual information, Delirium superimposed on dementia

## Abstract

**Background:**

Healthcare researchers often use multiple healthcare survey instruments to examine a particular patient symptom. The use of multiple instruments can pose some interesting research questions, such as whether the outcomes produced by the different instruments are in agreement. We tackle this problem using information theory, focusing on mutual information to compare outcomes from multiple healthcare survey instruments.

**Methods:**

We review existing methods of measuring agreement/disagreement between the instruments and suggest a procedure that utilizes mutual information to quantitatively measure the amount of information shared by outcomes from multiple healthcare survey instruments. We also include worked examples to explain the approach.

**Results:**

As a case study, we employ the suggested procedure to analyze multiple healthcare survey instruments used for detecting delirium superimposed on dementia (DSD) in community-dwelling older adults. In addition, several examples are used to assess the mutual information technique in comparison with other measures, such as odds ratio and Cohen’s kappa.

**Conclusions:**

Analysis of mutual information can be useful in explaining agreement/disagreement between multiple instruments. The suggested approach provides new insights into and potential improvements for the application of healthcare survey instruments.

## Background

Numerous healthcare survey instruments exist to identify or evaluate individual health status. Many are used to diagnose a particular symptom, serving as a diagnostic survey instrument. Since such diagnostic survey instruments are noninvasive, several instruments can be used on the same patient. Multiple healthcare survey instruments may be used to corroborate a preliminary conclusion when two or more instruments are used to target a particular phenomenon. Techniques for interpreting diagnostic results collectively can be found in several studies [1–3]. For example, by utilizing the area below a receiver operating characteristic (ROC) curve, one can combine multiple results to increase diagnostic accuracy [2]. It is also possible to examine the potential benefits of adding another diagnostic result onto the original results [1]. Generally, a healthcare researcher uses the reference standard instrument (gold standard when error-free) to adequately explain a patient’s true health status or to validate newly developed instruments. In such instances, diagnostic results can be combined to test the level of agreement with a reference standard and validate a newly developed diagnostic instrument [4]. To summarize, the use of multiple instruments suggests some interesting research questions, such as how outcomes examined by different instruments can be interpreted collectively.

In this paper we present a procedure that quantitatively compares and evaluates outcomes from multiple healthcare survey instruments using information theoretic measures, especially using mutual information. Conventionally, the odds ratio or Cohen’s kappa have been used to determine agreement/disagreement among assessments. The present novel procedure differentiates itself from existing techniques by using mutual information, which has been used in revealing association among results. Specifically, the core element of the procedure includes utilizing mutual information and checking the significance of the measure among multiple health care survey instruments. The advantage of this procedure over existing methods is that it provides well-bounded, responsive and easy-to-use measures. More importantly, this approach has great potential, allowing a broader, richer and more precise identification of meaningful information from the data collected from multiple survey instruments, when it is used with other information theoretic measures for an analysis. With the suggested approach, we compare multiple healthcare survey instruments used for detecting delirium.

Delirium is common and deadly in persons with dementia. It results in a more rapid downward trajectory in functional outcomes that can lead to institutionalization. Therefore, delirium must be detected quickly in order to prevent further functional decline and high healthcare costs associated with institutionalization. Family caregivers, because of their close relationship to the person with dementia, are in an excellent position to detect delirium in the home environment. In light of this, we aim to use the suggested approach to validate an assessment which was developed for family members to administer at their home. In addition, we applied the suggested approach to the other external studies for comparison purposes.

The layout of this paper is as follows. In the ‘Methods’ section, we review existing measures to quantitatively measure agreement/disagreement in outcomes from multiple instruments, and suggest mutual information as possible measure for this purpose. To this end, we suggest a procedure to compare with multiple instruments that utilizes mutual information. In the ‘Results’ section we use the suggested framework to compare and analyze a number of instruments that were used in a pilot study for detecting delirium. In addition, we apply the suggested approach to other studies that compare multiple healthcare survey instruments. Finally, through several illustrations, we compare mutual information with other competing measures that have been used in conventional studies. The ‘Discussion’ section considers a validity of the FAM-CAM used in the pilot study and offers discussion of some of the benefits of mutual information, exploring the applicability and limitations of the suggested approach.

## Methods

Before we review existing measures, we first distinguish ‘association’ from ‘agreement/disagreement.’ An association among data can be interpreted as dependency among the data. Dependency exists when one element of the data changes and one or more other elements of data also change. Even if the change is opposite, we still say that dependency exists among the data, since one change affects the other changes (this is sometimes referred to as ‘negative association’). In this paper, we restrict the use of the term agreement (disagreement) to positive association (negative association) and vice versa.

### Existing measures

For a straightforward approach to evaluating congruency of data, a raw agreement index can be used. This index can be obtained by calculating the portion of agreement sets over the entire sets. Level of agreement can also be calculated using Cohen’s kappa coefficient, which measures the level of agreement between two raters by using the term of chance agreement [5]. Cohen’s kappa coefficient can be expressed as shown in Eq. 1.

1 $$ \kappa =\frac{P(a)-P(e)}{1-P(e)} $$

where *P(a)* refers to the observed probability of agreement while *P(e)* is the expected probability of agreement. Although Cohen’s kappa is widely used, it is only valid in the case of two independent raters [6]. For multiple raters, Fleiss’ kappa can be considered for evaluating agreement, but it provides relatively weak evidence on the significance level [7]. The results from both Cohen’s kappa and Fleiss’ kappa can be difficult to apply across studies without an error generation model [8].

As another candidate measure, we focused on association analyses and the related tools that explain relationships among various types of data. Tan et al. compare many association measures to reveal the specific characteristics of each measure [9]. Of such measures, odds ratio, which is one of the most popular association measures, evaluates the strength of association between the two data values. The odds ratio of a 2 by 2 contingency table, for example, can be expressed as shown in Eq. 2.

2 $$ Odds\kern0.5em ratio=\frac{P\left({x}_{11}\right)P\left({x}_{22}\right)}{P\left({x}_{12}\right)P\left({x}_{21}\right)} $$

where *P(x*_*ij*_*)* is the probability of the cell whose row is i and column is j in a contingency table. Since odds ratio is easy to calculate, it is widely used to explain the level of association among the data, especially when validating alternative medical treatments. Odds ratio characterizes positive association (above 1), and negative association (below 1) among data.

### Mutual information and local mutual information

Mutual information measures the amount of shared information between two instruments as shown in Eq. 3 [10].

3 $$ I\left( Instrument\kern0.5em 1;\kern0.5em  Instrument\kern0.5em 2\right)={\sum}_{i,j}P\left({x}_{ij}\right)\kern0.5em { \log}_2\frac{P\left({x}_{ij}\right)}{P\left({x}_{i\cdot}\right)P\left({x}_{\cdot j}\right)} $$

where i and j stand for Instrument 1 and Instrument 2. Conventionally, mutual information has been used to measure the level of association among data. Some association measures can explain the direction of association, whether positive (agreement) or negative (disagreement). Mutual information itself, however, does not show direction of association, and it has been used to explain only the level of association, regardless of direction. Interestingly, recent studies suggest that local mutual information can be used to explain the level not only of association, but of agreement among the data [8, 11].

Theoretically, mutual information is the sum of local mutual information, which is $P\left({x}_{ij}\right)\kern0.5em { \log}_2\frac{P\left({x}_{ij}\right)}{P\left({x}_{i\cdot}\right)P\left({x}_{\cdot j}\right)}$. Such local mutual information is often regarded as an important measure and is used for information retrieval [12]. More importantly, some sets of local mutual information offer a quantitative measure of the level of agreement, beyond explaining association among the data. From a theoretical perspective, the use of mutual information as a measure of agreement can be beneficial as compared to other similar measures used for inter-rater reliability. First, it can be easily calculated without an error generation model [8], so it does not require any additional assumptions when applied across studies. Moreover, it can be approximated to a chi-square statistic, which enables us to statistically measure the level of significance of the mutual information obtained [13, 14]. Also, since mutual information is an information theoretical measure, it can be used along with other useful information theoretical measures, such as relative entropy or conditional entropy, thereby enhancing the potential for interpreting the data. A recent study utilizes mutual information and conditional entropy to extract “novel” information from medical data [15].

### Proposed procedure: comparison of two diagnostic instruments

**Fig. 1:**
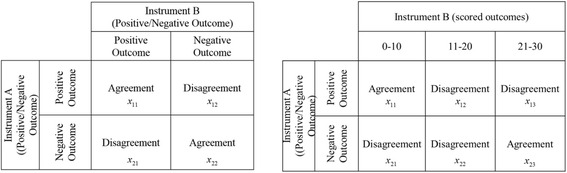
Examples of a contingency table

The proposed procedure for comparing two diagnostic instruments is summarized as shown in Table 1. The first step is to design a contingency table and collect data using the table. Then, the next step is to determine which cells in the table explain agreement or disagreement among the data. For convenience purposes, the cell explaining agreement (or disagreement) among the data is defined as an agreement section (or, disagreement section, respectively). Each category of each instrument should contain at least one agreement section and one disagreement section assigned in the table. The matrix shown on the left in Fig. 1 has two agreement sections (Positive-Positive, Negative-Negative) and two non-agreement sections (Positive–negative, Negative–positive). Additionally, one could assign “partial” agreement (or disagreement) sections by imposing a weight (ranging from 0 to 1) on each section [16].

**Table 1:** A procedure for comparing two instruments

Step 1. Design a contingency table and collect data using the table
Step 2. Measure mutual information
Step 3. Determine significance of the mutual information
Step 4. Check the sum of the local mutual information on agreement section

Once a contingency table is organized, we then measure the amount of shared information among the instruments by calculating mutual information, as we do for conventional association analysis. As mentioned earlier, mutual information consists of linear terms representing local mutual information. Using local mutual information, we can determine the level of agreement among different instruments. It can be expressed as follows;

4 $$ {I}_{agreement}\left( Inst.\kern0.5em 1;\kern0.5em  Inst.\kern0.5em 2\right)={\sum}_{\left(i,j\right)\in A}P\left({x}_{ij}\right){ \log}_2\frac{P\left({x}_{ij}\right)}{P\left({x}_{i\cdot}\right)P\left({x}_{\cdot j}\right)} $$

5 $$ {I}_{disagreement}\left( Inst.\kern0.5em 1;\kern0.5em  Inst.\kern0.5em 2\right)={\sum}_{{\left(i,j\right)}_{\in_D}}P\left({x}_{ij}\right){ \log}_2\frac{P\left({x}_{ij}\right)}{P\left({x}_{i\cdot}\right)P\left({x}_{\cdot j}\right)} $$

where *A* stands s and *D* stands for a set of disagreement sections between the two instruments (expressed as Inst.1 (Inst.2) representing instrument 1 (instrument 2, respectively)). By convention, $0{ \log}_2\frac{0}{p}=0$ and $p{ \log}_2\frac{p}{0}=\infty$, which is *p* ≠ 0.

In order to measure the significance of mutual information, we can use a technique for approximating sample mutual information. In general, over many trials, twice the sample mutual information can be approximated as a *χ*^2^ distribution, as shown in Eq. 6 [14].

6 $$ 2n{\displaystyle {\sum}_{i=1}^m{\sum}_{j=1}^nP\left({x}_{ij}\right){ \log}_e}{\frac{p\left({x}_{ij}\right)}{P\left({x}_{i\cdot}\right)P\left({x}_{\cdot j}\right)}}^{\sim }{\chi}^2\left(\nu \right) $$

where *n* is the size of the sample. If the base of the logarithm of Eq. 6 is changed from *e* to 2, then the mutual information term can be expressed as $2n\cdot \ln 2\cdot {\displaystyle {\sum}_{i=1}^m{\displaystyle {\sum}_{j=1}^nP\left({x}_{ij}\right){ \log}_2}}\frac{P\left({x}_{ij}\right)}{P\left({x}_{i\cdot}\right)P\left({x}_{\cdot j}\right)}$. It is known that this is distributed as a *χ*^*2*^ distribution with degree of freedom, *υ. υ* is determined by (the number of rows of a contingency table −1) × (the number of columns of a contingency table −1) [13]. From this conversion, the corresponding *χ*^*2*^ statistic can be calculated to determine the significance of the obtained mutual information in a frame of statistical testing. Also, we use p-value as a measure of strength of evidence, that is, significance of the mutual information.

To interpret the mutual information obtained, when a significant amount of mutual information is observed and the sum of local mutual information in the agreement sections is larger than that in the disagreement sections, we say that the two instruments are in agreement with each other. Similarly, if we observe a significant amount of mutual information and the sum of the local mutual information in the disagreement area is larger than that in the agreement area, we say the two instruments are in disagreement. Meanwhile, if we observe only a low level of mutual information from the outcomes, we cannot determine the level of agreement/disagreement of the outcomes. This situation falls under the inconclusive category, and is caused either by a lack of sufficient data to draw out associations or the existence of a truly independent relationship among the data.

### Example

For the better understanding of the proposed procedure, we show worked examples using fictitious data and illustrate step-by-step in Table 2. In this example, we compare two instruments, Instrument A and Instrument B, resulting in binary results, such as positive and negative, as shown in the left panel of Fig 1.

**Table 2:** Illustrative example of comparing two diagnostic instruments (Note: At Step 1, Numbers in agreement sections in each table are expressed in boldface)

Procedure	Scenario 1		Scenario 2		Scenario 3	
Data (${x}_{11}$, ${x}_{12}$, ${x}_{21}$, ${x}_{22}$)	(10, 5, 5, 20)		(5, 10, 20, 5)		(5, 10, 5, 20)	
Step 1 Contingency table	**10** 5	5 **20**	**5** 20	10 **5**	**5** 5	10 **20**
Step 2	${I_{agreement}}_{+\left(\frac{20}{40}\right){ \log}_2\frac{\left(\frac{20}{40}\right)}{\left(\frac{25}{40}\right)\left(\frac{25}{40}\right)}}^{=\left(\frac{10}{40}\right){ \log}_2\frac{\left(\frac{10}{40}\right)}{\frac{(15)}{40}\left(\frac{15}{40}\right)}}$		${I_{agreement}}_{+\left(\frac{5}{40}\right){ \log}_2\frac{\left(\frac{5}{40}\right)}{\left(\frac{15}{40}\right)\left(\frac{25}{40}\right)}}^{=\left(\frac{5}{40}\right){ \log}_2\frac{\left(\frac{5}{40}\right)}{\frac{(15)}{40}\left(\frac{25}{40}\right)}}$		${I_{agreement}}_{+\left(\frac{20}{40}\right){ \log}_2\frac{\left(\frac{20}{40}\right)}{\left(\frac{30}{40}\right)\left(\frac{25}{40}\right)}}^{=\left(\frac{5}{40}\right){ \log}_2\frac{\left(\frac{5}{40}\right)}{\frac{(15)}{40}\left(\frac{10}{40}\right)}}$	
Mutual information						
	${I_{disagreement}}_{+\left(\frac{5}{40}\right){ \log}_2\frac{\left(\frac{5}{40}\right)}{\left(\frac{15}{40}\right)\left(\frac{25}{40}\right)}}^{=\left(\frac{5}{40}\right){ \log}_2\frac{\left(\frac{5}{40}\right)}{\frac{(15)}{40}\left(\frac{25}{40}\right)}}$		${I_{disagreement}}_{+\left(\frac{20}{40}\right){ \log}_2\frac{\left(\frac{20}{40}\right)}{\left(\frac{25}{40}\right)\left(\frac{25}{40}\right)}}^{=\left(\frac{10}{40}\right){ \log}_2\frac{\left(\frac{10}{40}\right)}{\frac{(15)}{40}\left(\frac{15}{40}\right)}}$		${I_{disagreement}}_{+\left(\frac{5}{40}\right){ \log}_2\frac{\left(\frac{5}{40}\right)}{\left(\frac{10}{40}\right)\left(\frac{25}{40}\right)}}^{=\left(\frac{10}{40}\right){ \log}_2\frac{\left(\frac{10}{40}\right)}{\frac{(15)}{40}\left(\frac{30}{40}\right)}}$	
Step 3 Significance	Mutual information = 0.386-0.227 = 0.159 From (Eq. 6), 2*40*ln2*0.159 = 8.809 Highly significant (with *p <* 0.001)		Mutual information = -0.227 + 0.386 = 0.159 From (Eq. 6), 2*40*ln2*0.159 = 8.809 Highly significant (with *p <* 0.001)		Mutual information = 0.098-0.083 = 0.015 From (Eq. 6), 2*40*ln2*0.015 = 0.871 Less significant (with *p =* 0.35)	
Step 4 Local mutual information	*I*_*agreement*_ > *I*_*disagreement*_ and highly significant mutual information. Thus, agreement		*I*_*agreement*_ < *I*_*disagreement*_ and highly significant mutual information. Thus, disagreement		*I*_*agreement*_ > *I*_*disagreement*_ but very low mutual information observed. Thus, inconclusive	

## Results: analysis of assessments to detect delirium

As a case study, we analyzed the outcomes of a pilot study on the feasibility of enlisting family caregivers to electronically report delirium symptoms in patients with dementia [17]. The purpose of this pilot study was to prospectively explore the feasibility of engaging family caregivers to electronically report observations of delirium symptoms in community-dwelling older adults with dementia. This study also sought to describe agreement between family observations of delirium (Family Confusion Assessment Method [FAM-CAM]) and researcher assessments (Confusion Assessment Method [CAM]). Family caregivers accessed an electronic delirium assessment instrument via their personal computer or a study supplied smart phone daily to transmit data. There were 13 patient participants in this pilot study. All were Caucasian, mean age 80, sixty-nine percent were female and mean years of education was 11. Caregivers were adult children (*N* = 8), spouses (*N* = 4) and siblings (*N* = 1). Eight caregivers used their own personal computers and five used study supplied smart phones. The pilot study and consents were approved by the Penn State and University of Pennsylvania Institutional Review Board (IRB)s.

Delirium was operationally defined according to the validated CAM criteria and the Delirium Rating Scale (DRS-R-98). The CAM features are 1) acute onset and fluctuating course, 2) inattention, and either 3) disorganized thinking, or 4) altered level of consciousness [18]. The CAM is a standardized screening tool allowing persons without formal psychiatric training to quickly and accurately identify delirium. The FAM-CAM was developed as part of a larger cohort study as a means to detect delirium in elders; it relies on caregiver information to screen for the CAM features. While the FAM-CAM is based on the original CAM, there are differences between the two instruments. The health care professional administering the CAM and employing observational skills, assesses the four main features of delirium directly. In contrast, the FAM-CAM includes questions directed for the family member to help identify the cardinal signs of delirium as well as those sensitive to detect delirium (i.e., inattention, disorganized thinking, lethargy, disorientation, perceptual disturbances and inappropriate behavior/agitation). According to the diagnostic algorithm, delirium is identified if the patient shows the presence of acute onset, fluctuating course, inattention, and either the presence of disorganized thinking or an altered level of consciousness.

Theoretically, CAM and FAM-CAM share the same features for detecting delirium, so they are expected to produce identical outcomes. Practically, however, they are not identical in terms of content; the assessments have different structure and content, as appropriate to their original purposes. Compared to the CAM, the FAM-CAM paraphrases all contents of the CAM so that the assessment can be conducted easily by family caregivers. For this reason, some of the contents of FAM-CAM might be conveyed inaccurately to caregivers and fail to meet the original intentions of the CAM. Consequently, the validity of FAM-CAM should be checked in a rigorous way. Along with the CAM and FAM-CAM, other instruments designed to detect different aspects of a patient’s DSD were used in the pilot. Trained professionals administered instruments such as the Mini-Mental State Examination (MMSE), Clinical Dementia Rating (CDR), and the Delirium Rating Scale (DRS). In reality, CAM was used as the tool to detect delirium, while the other instruments were used to detect the severity of either dementia or delirium.

First, we compared overall outcomes of CAM and FAM-CAM using the suggested approach. In total, we collected 41 cases of CAM and FAM-CAM. Of 41 cases, 8 true positive and 31 true negative cases were observed. The detailed results are summarized in Table 3. From the contingency table, mutual information between CAM and FAM-CAM was calculated at 0.492. To test the significance of the mutual information obtained, we calculated a corresponding chi-square statistic by approximating the mutual information. As a result of the significance, the chi-square statistic was 27.977 with 1 degree of freedom, and highly significant, with *p <* 0.001. For comparison purposes, we note that the odds ratio was 248 and Cohen’s kappa was 0.858, both significant. For the next step, we tested agreement between the methods by comparing the sign of the local mutual information of agreement sections with that of non-agreement sections. The local mutual information from the agreement area (0.629) was found to be positive, while the local mutual information from the disagreement sections (−0.137) was found to be negative. Thus, we can conclude that the outcomes from CAM and FAM-CAM share high levels of information, and they were in agreement with each other. In other words, the outcomes from the two instruments show a high level of agreement.

**Table 3:** A contingency table for CAM and FAM-CAM (Numbers in agreement sections expressed in boldface)

		CAM		
		Positive	Negative	Total
FAM-CAM	Positive	**8 (Agreement)**	1 (Disagreement)	9
	Negative	1 (Disagreement)	**31 (Agreement)**	12
	Total	9	32	41

Similarly, we checked the level of agreement of each primary feature between CAM and FAM-CAM. In terms of the significance of the mutual information, we observed a high level of significance between CAM and FAM-CAM in the case of feature 1 (acute onset and fluctuating courses) and feature 3 (disorganized thinking). In addition, we observed a positive amount of local mutual information on the agreement area for all features. Therefore, we can conclude that the two instruments are in agreement in terms of features 1 and 3. For feature 2, although we observed that the outcomes for the feature agreed, we also observed that its significance level is relatively low, compared to features 1 and 3. Meanwhile, we measured quite a low level of mutual information on feature 4 (altered level of consciousness). That is, this situation falls into the inconclusive category. In this case, odds ratio and Cohen’s kappa was also measured as insignificant. This can be caused by either independent outcomes or a shortage of data on the corresponding feature, so further investigation with a domain expert is necessary.

We also checked whether different access to each instrument resulted in different outcomes. In the pilot, we provided both trained professionals and family caregivers with an Internet-based instrument. For this, we built a web-based application system, called e-Care for Eldercare, and allowed all examiners access to the system to use the instrument electronically. To add flexibility to the system, we also allowed some of the family caregivers without an Internet-connected environment to access the system via a smartphone. Unfortunately, the smartphone used in the study had a small touch screen (2.4 inches) that made it difficult to view clearly and operate properly, so the family caregivers frequently entered incorrect information by mistake. We hypothesized that such usability issues might cause differences in the outcomes of CAM and FAM-CAM, and we compared the groups using mutual information. Overall, although the outcomes of CAM and FAM-CAM between the two groups are in agreement, mutual information measured from smartphone group was lower compared to overall group. As another example, we also analyzed the study of Naylor, in which CAM and FAM-CAM were also compared [19]. That study used a paper-based FAM-CAM administered by nurses, so the subjects, examiners, and experimental settings were different from those of our pilot study. Naylor’s study also showed a high level of agreement among instruments. Table 4 shows local mutual information on both agreement and disagreement sections of all comparisons described here, along with odds ratio and Cohen’s kappa coefficient for comparison purposes.

**Table 4:** Comparison of FAM-CAM with other instruments

		Comparison using local mutual information					
Pair no.	Comparisons of FAM-CAM with	*I*_*agreement*_	*I*_*disagreement*_	Mutual information	Result	Odds ratio	Kappa
1	CAM	0.629	-0.137	0.492	Agreement (*p*-value < 0.01)	248	0.858
2	Feature of CAM: Acute onset & fluctuating courses	0.356	-0.177	0.179	Agreement (*p*-value < 0.01)	17.6	0.424
3	Feature of CAM: Inattention	0.196	-0.148	0.048	Agreement (*p*-value < 0.1)	3.125*	0.237
4	Feature of CAM: Disorganized thinking	0.320	-0.116	0.204	Agreement (*p*-value < 0.01)	∞**	0.346
5	Feature of CAM: Altered level of consciousness	-0.022	0.025	0.003	Inconclusive	0.65*	-0.051*
6	FAM-CAM on a group using smart phones	0.519	-0.245	0.274	Agreement (*p*-value < 0.05)	18***	0.607
7	FAM-CAM from other study	0.551	- 0.099	0.452	Agreement (*p*-value < 0.01)	∞**	0.805

(Note: *insignificant at confidence level = 90 %, **Infinity since one of the cells contains 0, ***insignificant at confidence level = 95 %, but significant at level = 90 %,)

In addition to the CAM, trained professionals of the pilot team also implemented a series of instruments, including the Delirium Rating Scale (DRS) and the Mini-Mental State Examination (MMSE). Of these, DRS is an assessment for measuring level of delirium and was used repeatedly on individual patients, along with the CAM. Theoretically, CAM, DRS, and FAM-CAM share a similar diagnostic purpose, since they were all designed to assess delirium symptoms. Both CAM and FAM-CAM provide binary results on the presence of delirium, while DRS uses numerical scale numbers indicating the severity of delirium. Thus, we can conjecture that the three different instruments will provide similar results for detecting delirium.

In our analysis, we use cut-off information for dividing the results from DRS into two categories [20]. The usual cut-off scores for severity in the DRS are 16 or 17 out of 39, and we followed this rule when categorizing DRS results. We found that both cut-off scores (16 or 17) are not significant to change the final results. Table 5 shows the mutual information observed from the three possible pairs: (CAM, DRS), (CAM, FAM-CAM) and (FAM-CAM, DRS); the analysis showed a considerable amount of mutual information in the direction of agreement for the pairs (CAM, DRS) and (CAM, FAM-CAM), while the (FAM-CAM, DRS) pair failed to show enough mutual information to conclude as to agreement or disagreement.

**Table 5:** Comparison result of each pair of instruments

		Comparison using local mutual information					
Pair no.	Pair	${I}_{agreement}$	${I}_{disagreement}$	Mutual information	Result	Odds ratio	Kappa
8	(CAM, FAMCAM)	0.397	-0.173	0.224	Agreement (*p*value < 0.01)	25.333	0.575
9	(FAMCAM, DRS)	-0.090	0.115	0.025	Inconclusive (*p* = 0.335)	0.381*	-0.128*
10	(DRS, CAM)	0.275	-0.176	0.099	Agreement (*p*-value < 0.1)	5.833	0.349

(Note: *insignificant at confidence level = 90 %)

## Results: analysis of assessments from other case studies

We apply mutual information to several other studies that compare multiple healthcare survey instruments [21–23], and summarize the results in Table 6. As candidate analytical measures, we consider odds ratio, Cohen’s kappa coefficient and the local mutual information technique proposed here. The studies considered here do not include common measures/procedures that could be used to explain either agreement or disagreement among the compared instruments. Some of these studies tested the contingency table using chi-square only, which explains the significance of association, but is not suitable for explaining agreement/disagreement. Some of the studies used measures that consider only partial information (such as sensitivity, specificity or McNemar’s test) in a contingency table, which could lead to a biased analysis; odds ratio, Cohen’s kappa or mutual information can, on the other hand, capture the whole contingency table.

**Table 6:** Agreement/disagreement between two instruments using three measures

Data set	Measure		
	Odds ratio	Cohen’s kappa	Local mutual information
(Table 2 in Shulman et al. 1986) Clock exam and MMSE^a^	15.6	0.493	Agreement (0.422 in *I*_*agreement*_, -0.212 in *I*_*disagreement*_)
(Figure 1 in Russell et al. 2012) BDI^b^ + CDRS_R^c^ and ICD-10^d^	∞**	0.693	Agreement (0.18 in *I*_*agreement*_, -0.03 in *I*_*disagreement*_)
(Figure 1 in Russell et al. 2012) CDRS_R and ICD-10	0.311*	-0.015*	Inconclusive ( -0.018 in *I*_*agreement*_, 0.024 in *I*_*disagreement*_)
(Table 4 in Seago 2002) Score scheme comparison	4	0.265	Agreement ( 0.152 in *I*_*agreement*_, -0.107 in *I*_*disagreement*_)

(Note: * insignificant at confidence level = 95 %, ** Infinity since one of the cells contains 0, ^a^Mini mental state examination, ^b^Beck Depression Inventory, ^c^Children’s Depression Rating Scale-Revised, ^d^International Classification Disease – 10 clinical interview)

## Results: illustrative comparisons among measures

**Fig. 2:**
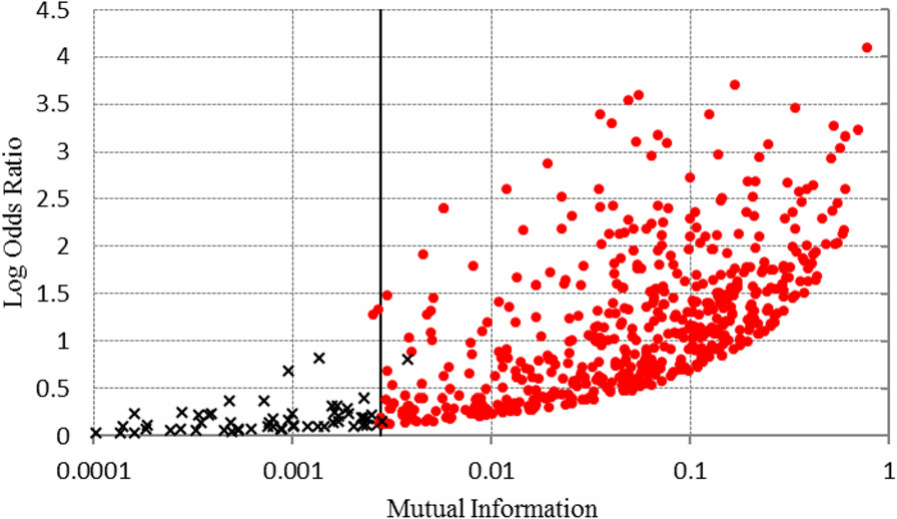
Comparison between log odds ratio and mutual information representing agreement. Insignificant odds ratios are marked as ‘x’ while significant ones are marked as ‘o’. The line representing significance limit (0.00277) of mutual information given the sample population has been inserted. Legend: Point ‘x’ : insignificant odds ratio. Point ‘o’ : significant odds ratio

In this section, we choose odds ratio and Cohen’s kappa coefficient, as representative competing measures, and compare them with mutual information in terms of measuring agreement/disagreement, with some illustrations. In Fig. 2, we compare mutual information with odds ratio using sample data on a plot. Note that we use logarithmic odds ratio instead of using raw odds ratio due to a scale issue. From the figure, we see that the overall trend of the odds ratio is well aligned with that of mutual information in terms of 1) agreement between data, and 2) insignificant values; as the odds ratio increases, mutual information also increases (Pearson correlation = 0.648). In most cases, if the compared data results in insignificant odds ratio, then it also results in insignificant value when measuring mutual information. In terms of intensity, however, the relative difference among odds ratios ranges up to 10,000 times in this illustration, which is poorly scaled up, while mutual information is well-bounded and does not exceed 1, which is a theoretical upper limit of each measure. Theoretically, odds ratio does not have an upper limit, whereas mutual information and Cohen’s kappa are well-bounded. From this illustration, we can conjecture that the odds ratios are highly likely to exaggerate the intensity of agreement as compared with the other two measures.

**Fig. 3:**
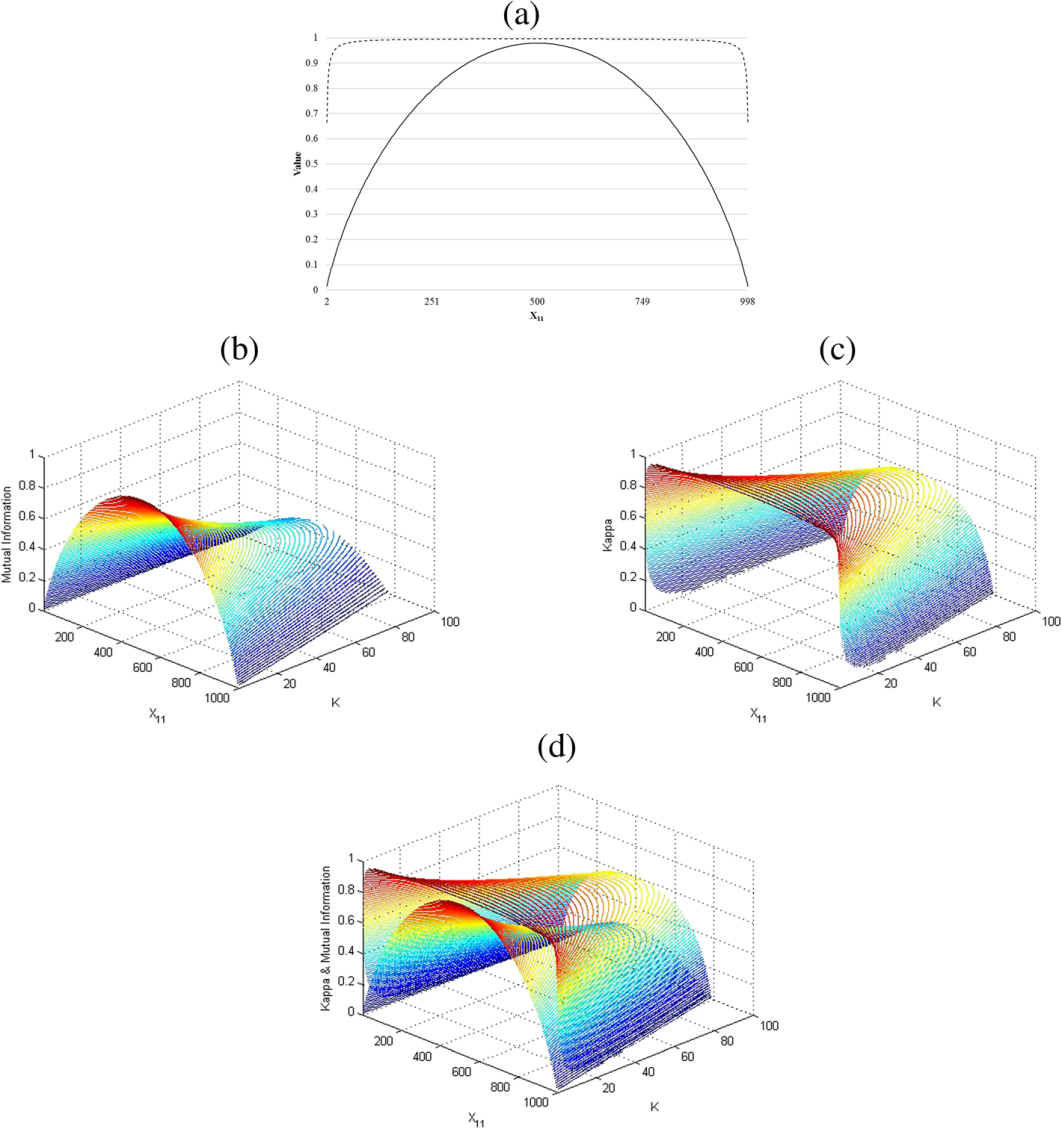
**a** comparison between mutual information and Cohen’s kappa coefficient when k=1, **b** mutual information when k > 0, **c** Cohen’s kappa when k > 0, **d** comparison of mutual information and Cohen’s kappa when k > 0. Legend: *dotted line* : Kappa. *sold line*: Mutual Information

Like mutual information, Cohen’s kappa appears to be a good candidate to measure the intensity of agreement. As the Cohen’s kappa increases, mutual information also increases (Pearson correlation = 0.831). However, compared to mutual information, Cohen’s kappa tends to result in high value regardless of the type of agreement, as long as the total amount of agreement is high. As illustration, consider the contingency table shown in the left of Fig. 1. We increase x_11_ from 1 to 1000 and decrease x_22_ from 1000 to 1 by 1, and set other cells, x_12_ and x_21_ to k > 0 and measure both mutual information and Cohen’s kappa for each combination of x_11,_ x_12,_ x_21,_ x_22_. That is, we change the ratio between positive-positive type agreement and negative-negative agreement, as the total number of agreements is maintained. Intuitively, we can conjecture that the level of agreement of data increases as x_11_ approaches x_22_, and the level of agreement of data will be the highest when x_11_ reaches x_22_. From Fig. 3(a), both mutual information and Cohen’s kappa show the highest agreement at x11 = x22 = 499 when k is set as 1 (Cohen’s kappa = 0.996 and mutual information = 0.979), but the trend approaching the highest value turns out to be different; Cohen’s kappa tends to quickly approach the highest value and then slowly converges before hitting the peak, while mutual information increases smoothly to the highest value as x_11_ and x_22_ approach equality. In other words, we can say that Cohen’s kappa tends to maintain high value regardless of the ratio of each agreement section, as long as the total amount of agreement overwhelms disagreement. On the other hand, mutual information emphasizes the situation where the amounts of agreement, positive-positive and negative-negative, approach each other.

We increase k from 1 to 100, which is the amount of disagreement, to see if the trend seen in Fig. 3(a) persists. We plot the changes of mutual information and Cohen’s kappa with the changes of k as shown in Fig. 3(b) and (c). Both figures show that the amount of agreement is maximized when x_11=_x_22_ for every k, but decreases as the amount of disagreement (k) increases. This confirms the trend shown in Fig. 3(a). We combine Fig. 3(b) and (c) into Fig. 3(d) for comparison.

## Discussion

The results from CAM and FAM-CAM show moderate or high levels of agreement in terms of overall results, featured levels, and platform/study. In most cases, mutual information and the other measures considered here result in the same results in terms of agreement/disagreement. Therefore, we can conclude that FAM-CAM, an adapted version of CAM, is a valid instrument for detecting delirium when used by family members.

During the comparisons, we examined the amount of information from each agreement and disagreement section separately, using the suggested approach. In some cases, we observed that there exists a weak level of agreement between the instruments compared. ‘Weak’ agreement can occur when there is a low amount of local mutual information from either agreement sections or disagreement sections. CAM and FAM-CAM (pair 1), for example, showed a high level of local mutual information on the agreement sections, compared to that from the disagreement sections (0.629 from agreement sections vs. -0.137 from disagreement sections). In the comparison of CAM and FAM-CAM in terms of the ‘Inattention’ feature (pair 3), however, the amount of local mutual information from the disagreement sections is measured at −0.148, while that of agreement sections is measured at only 0.196, resulting in 0.048 as mutual information, which represents a low level of agreement compared to pair 1. Thus, we conjecture that the level of agreement of pair 3 results in a relatively weak agreement, due to the low level of local mutual information from its agreement sections. In other words, FAM-CAM did not secure enough information to explain agreement with CAM in terms of the ‘Inattention’ feature, thereby leading to the need to further clarify FAM-CAM questions related to this feature. Meanwhile, although pair 8 (comparison with CAM and FAM-CAM) and pair 10 (comparison with CAM and DRS) show similar levels of local mutual information from disagreement sections (−0.173 and −0.176), pair 8 shows greater agreement due to the greater amount of local mutual information (0.397) from agreement sections compared to that of pair 10 (0.275). Thus, we conjecture that FAM-CAM shows better performance in terms of explaining agreement compared to DRS.

As another example, although the outcomes of CAM and FAM-CAM of smartphone groups is in agreement, mutual information measured from the group was lower compared to the overall group; pair 1(overall group) and pair 6 (smartphone group) show a weak levels of local mutual information from both agreement sections and disagreement sections. In this case, we can conclude that different access to the system affects the overall outcomes in terms of both agreement and disagreement type, thereby needing to improve usability of smartphone environment overall (e.g. user interface). In sum, with the suggested approach, we were able to explain why the comparison results in a weak level of agreement, by referring to local mutual information observed from both agreement sections and disagreement sections. Meanwhile, odds ratio and Cohen’s kappa do not have the ability to explain why such weak agreement between the instruments occurs.

Through a series of illustrations, we also show that mutual information offers various benefits over other competing measures. First, odds ratio sometimes scales up poorly. When we analyze the data from the case study for detecting delirium, we see that the odds ratio is highly likely to exaggerate the level of agreement (248 from the comparison of CAM and FAM-CAM) and sometimes cannot be measured properly (infinity due to 0 in the data). Meanwhile, both Cohen’s kappa and mutual information are measured within a reasonable bound for most of the studies. In addition, we see that Cohen’s kappa is affected only by the total amount of agreement between the instruments. Mutual information, on the other hand, can be affected not only by the total amount of agreement, but also by the amount of each agreement type. In our case study, for example, for pair 1, Cohen’s kappa coefficient was measured at 0.858, which is fairly high. In that case, mutual information was measured at 0.492. We used Table 3 as data for measuring those measures; 39 observations from the agreement sections produce 8 positive-positive agreements and 31 negative-negative agreements. As a hypothetical situation, if there were an almost even number of observations from each agreement (20 positive-positive and 19 negative-negative), the mutual information would be increased to 0.718 (an increase of 45.9 %) while Cohen’s kappa is would increase only slightly, to 0.902 (an increase of 5.1 %). In other words, mutual information places more weight on the evenness of the amount of agreement evidence, as compared to Cohen’s kappa. Consequently, we conjecture that the suggested approach is more capable of providing separate information both agreement and disagreement sections, a well-scaled measure as compared to odds ratio, and adequate-responsive measure to the agreement type.

Some limitations of using mutual information in comparing instruments are as follows. First, applying mutual information could be demanding in cases of three or more instruments, since the concept of mutual information only applies to comparison of two instruments. Meanwhile Fleiss’ kappa, an extended form of Cohen’s kappa, could be used to measure agreement level in this situation. Another limitation is that use of mutual information assumes that each instrument to be compared should have the similar diagnostic purpose. In our case study, the team also administered the Mini-Mental State Examination (MMSE), which is used to measure the level of a patient’s cognitive impairment. A severe level of cognitive impairment may be due not only to some level of delirium, but also to the severity of dementia. In this sense, the diagnostic purpose of the MMSE is somewhat different from those of the CAM, FAM-CAM, and DRS, which are dedicated to examining a patient’s delirium. Since mutual information can be applied only for comparing instruments with similar diagnostic purpose, its usage could be limited in comparing MMSE and other instruments for delirium. Our future research will primarily focus on how we can compare three or more diagnostic instruments and different instruments that do not share the same diagnostic purpose. For this, we need to extend the suggested approach and explore other information theoretic approaches.

## Conclusion

In this paper, we suggest a procedure for comparing multiple healthcare survey instruments using mutual information, an information theoretic approach. Mutual information is used to measure the amount of information shared among the outcomes from multiple instruments. With the suggested procedure, we explain agreement/disagreement between the instruments used in several studies and compare with other competing measures to show the benefits of the mutual information. Our suggested approach is more capable of providing separate information with both agreement and disagreement existing in the data, a well-scaled measure and adequate-responsive measure to the agreement type compared to other competing measures. We also mentioned an instrument can be further improved by referring to the information measured from agreement and disagreement. We believe the use of this approach will provide a reliable approach to evaluate agreement/disagreement of outcomes from multiple instruments and may also offer clues to improving healthcare survey instruments.

## Abbreviations

CAM, Confusion Assessment Method; CDR, Clinical Dementia Rating; DRS, Delirium Rating Scale; DSD, Delirium superimposed on dementia; FAM-CAM, a family version of CAM; MMSE, Mini-Mental State Examination
